# 2-Amino-5-methyl­pyridinium trifluoro­acetate

**DOI:** 10.1107/S1600536812045291

**Published:** 2012-11-10

**Authors:** Kaliyaperumal Thanigaimani, Abbas Farhadikoutenaei, Nuridayanti Che Khalib, Suhana Arshad, Ibrahim Abdul Razak

**Affiliations:** a School of Physics, Universiti Sains Malaysia, 11800 USM, Penang, Malaysia

## Abstract

In the title salt, C_6_H_9_N_2_^+^·C_2_F_3_O_2_^−^, the F atoms of the anion are disordered over two sets of sites, with refined occupancies in a ratio of 0.505 (17):0.495 (17). In the crystal, cations and anions are linked *via* N—H⋯O hydrogen bonds, forming *R*_2_^2^(8) ring motifs. The ionic units are linked into a two-dimensional network parallel to (100) by N—H⋯O and weak C—H⋯O hydrogen bonds. The crystal structure is further stabilized by weak C—H⋯F hydrogen bonds, resulting in a three-dimensional network.

## Related literature

For background to the chemistry of substituted pyridines, see: Pozharski *et al.* (1997 ▶); Katritzky *et al.* (1996 ▶). For details of hydrogen bonding, see: Jeffrey & Saenger (1991 ▶); Jeffrey (1997 ▶); Scheiner (1997 ▶). For hydrogen-bond motifs, see: Bernstein *et al.* (1995 ▶). For standard bond-length data, see: Allen *et al.* (1987 ▶). For the stability of the temperature controller used for the data collection, see: Cosier & Glazer (1986 ▶). For a related structure, see: Rodrigues *et al.* (2001 ▶).
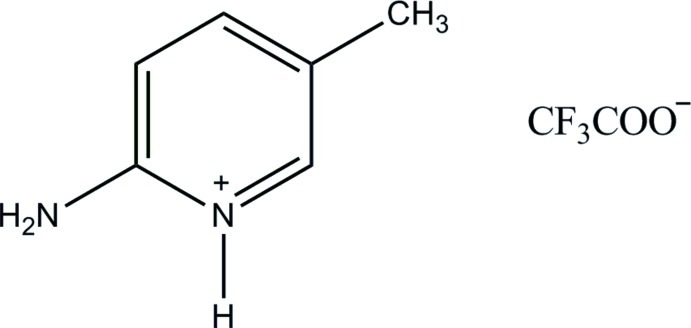


## Experimental

### 

#### Crystal data

C_6_H_9_N_2_^+^·C_2_F_3_O_2_^−^
*M* *_r_* = 222.17
Orthorhombic, 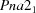

*a* = 18.725 (4) Å
*b* = 4.6256 (10) Å
*c* = 11.319 (2) Å
*V* = 980.4 (3) Å^3^
*Z* = 4
Mo *K*α radiation
μ = 0.15 mm^−1^
*T* = 100 K
0.54 × 0.29 × 0.11 mm

#### Data collection

Bruker SMART APEXII DUO CCD area-detector diffractometer
Absorption correction: multi-scan (*SADABS*; Bruker, 2009 ▶) *T*_min_ = 0.926, *T*_max_ = 0.985
12012 measured reflections
3216 independent reflections
2627 reflections with *I* > 2σ(*I*)
*R*_int_ = 0.041

#### Refinement

*R*[*F* ^2^ > 2σ(*F* ^2^)] = 0.046
*wR*(*F* ^2^) = 0.114
*S* = 1.07
3216 reflections
177 parameters
1 restraint
H atoms treated by a mixture of independent and constrained refinement
Δρ_max_ = 0.23 e Å^−3^
Δρ_min_ = −0.30 e Å^−3^
Absolute structure: Flack (1983 ▶), 1368 Friedel pairs
Flack parameter: −0.1 (7)

### 

Data collection: *APEX2* (Bruker, 2009 ▶); cell refinement: *SAINT* (Bruker, 2009 ▶); data reduction: *SAINT*; program(s) used to solve structure: *SHELXTL* (Sheldrick, 2008 ▶); program(s) used to refine structure: *SHELXTL*; molecular graphics: *SHELXTL*; software used to prepare material for publication: *SHELXTL* and *PLATON* (Spek, 2009 ▶).

## Supplementary Material

Crystal structure: contains datablock(s) global, I. DOI: 10.1107/S1600536812045291/lh5549sup1.cif

Structure factors: contains datablock(s) I. DOI: 10.1107/S1600536812045291/lh5549Isup2.hkl

Supplementary material file. DOI: 10.1107/S1600536812045291/lh5549Isup3.cml

Additional supplementary materials:  crystallographic information; 3D view; checkCIF report

## Figures and Tables

**Table 1:** Hydrogen-bond geometry (Å, °)

*D*—H⋯*A*	*D*—H	H⋯*A*	*D*⋯*A*	*D*—H⋯*A*
N1—H1*N*1⋯O2	0.98 (3)	1.75 (3)	2.7281 (19)	177 (2)
N2—H2*N*2⋯O1	0.95 (3)	1.92 (3)	2.865 (2)	173 (2)
N2—H1*N*2⋯O2^i^	0.86 (3)	1.99 (3)	2.8347 (18)	167 (3)
C3—H3*A*⋯F2^ii^	0.95	2.51	3.429 (6)	164
C5—H5*A*⋯O1^iii^	0.95	2.27	3.1910 (19)	162

Symmetry codes: (i) 
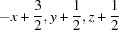
; (ii) 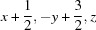; (iii) 
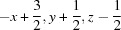
.

**Fig. 1.**
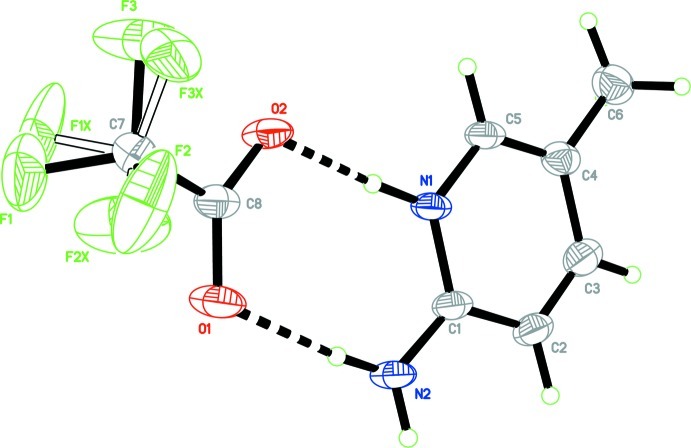
The molecular structure of the title compound, showing 50% probability displacement ellipsoids and the atom-numbering scheme. Both disorder components are shown.

**Fig. 2.**
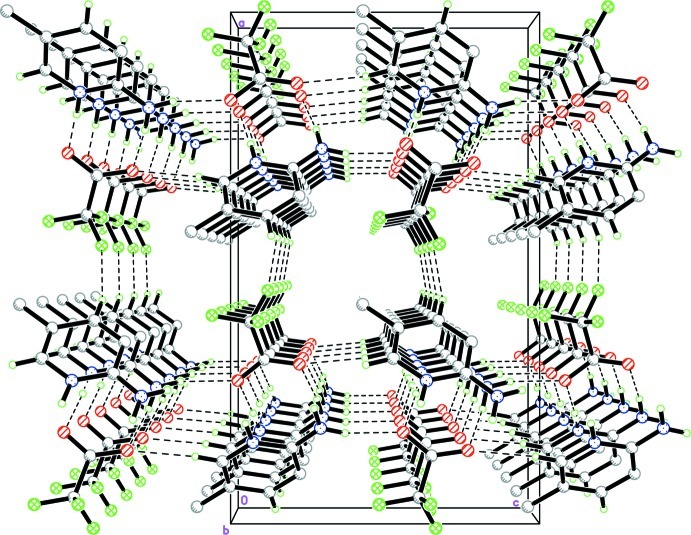
The crystal packing diagram of the title compound. Only major disorder component is shown. Hydrogen bonds are shown as dashed lines.

## supplementary crystallographic information

### Comment

Pyridine and its derivatives play an important role in heterocyclic chemistry (Pozharski *et al.*, 1997; Katritzky *et al.*, 1996). They are often involved in hydrogen-bond interactions (Jeffrey & Saenger, 1991; Jeffrey, 1997; Scheiner, 1997). Trifluoroacetic acid is a very strong carboxylic acid, easily volatile, and used for protein purification. An example of a crystal structure of a trifluoroacetate salts has been reported (Rodrigues *et al.*, 2001). In order to study potential hydrogen bonding interactions the crystal structure determination of the title compound (I) was carried out.

The asymmetric unit (Fig. 1) contains one 2-amino-5-methylpyridinium cation and one trifluoroacetate anion. The F atoms of the anion are disordered over two sets of sites, with occupancies of 0.505 (17) and 0.495 (17). In the 2-amino-5-methylpyridinium cation, a wider than normal angle [C1—N1—C5 = 122.77 (14)°] is subtended at the protonated N1 atom. The 2-amino-5-methylpyridinium cation is essentially planar, with a maximum deviation of 0.016 (2) Å for atom N2. The bond lengths (Allen *et al.*, 1987) and angles are normal.

In the crystal (Fig. 2), the cations and anions are linked *via* N—H···O hydrogen bonds to form *R*_2_^2^(8) ring motifs (Bernstein *et al.*, 1995). The ionic units are linked into a two-dimensional network parallel to (100) by N2—H1N2···O2^i^ and C5—H5A···O1^iii^ hydrogen bonds (symmetry codes in Table 1). The crystal structure is further stabilized by C3—H3A···F2^ii^ hydrogen bonds, resulting in a three-dimensional network.

### Experimental

To a hot methanol solution (20 ml) of 2-amino-5-methylpyridine (54 mg, Aldrich) was added a few drops of trifluoroacetic acid. The solution was warmed over a heating magnetic stirrer hotplate for a few minutes. The resulting solution was allowed to cool slowly at room temperature and crystals of the title compound (I) appeared after a few days.

### Refinement

The F atoms of the anion are disordered over two sets of sites, with occupancies of 0.505 (17):0.495 (17). Atoms H1N1, H1N2 and H2N2 were located in a difference Fourier maps and refined freely. The remaining hydrogen atoms were positioned geometrically [C–H= 0.95–0.98 Å] and were refined using a riding model, with *U*_iso_(H)=1.2 *U*_eq_(C) or 1.5*U*_eq_(methyl C). A rotating group model was used for the methyl group.

### Crystal data

**Table:**

C_6_H_9_N_2_^+^·C_2_F_3_O_2_^−^	*F*(000) = 456
*M**_r_* = 222.17	*D*_x_ = 1.505 Mg m^−^^3^
Orthorhombic, *P**n**a*2_1_	Mo *K*α radiation, λ = 0.71073 Å
Hall symbol: P 2c -2n	Cell parameters from 4200 reflections
*a* = 18.725 (4) Å	θ = 2.8–32.5°
*b* = 4.6256 (10) Å	µ = 0.15 mm^−^^1^
*c* = 11.319 (2) Å	*T* = 100 K
*V* = 980.4 (3) Å^3^	Plate, colourless
*Z* = 4	0.54 × 0.29 × 0.11 mm

### Data collection

**Table:**

Bruker SMART APEXII DUO CCD area-detector diffractometer	3216 independent reflections
Radiation source: fine-focus sealed tube	2627 reflections with *I* > 2σ(*I*)
Graphite monochromator	*R*_int_ = 0.041
φ and ω scans	θ_max_ = 32.7°, θ_min_ = 2.2°
Absorption correction: multi-scan (*SADABS*; Bruker, 2009)	*h* = −28→28
*T*_min_ = 0.926, *T*_max_ = 0.985	*k* = −6→6
12012 measured reflections	*l* = −16→17

### Refinement

**Table:**

Refinement on *F*^2^	Secondary atom site location: difference Fourier map
Least-squares matrix: full	Hydrogen site location: inferred from neighbouring sites
*R*[*F*^2^ > 2σ(*F*^2^)] = 0.046	H atoms treated by a mixture of independent and constrained refinement
*wR*(*F*^2^) = 0.114	*w* = 1/[σ^2^(*F*_o_^2^) + (0.0538*P*)^2^ + 0.1405*P*] where *P* = (*F*_o_^2^ + 2*F*_c_^2^)/3
*S* = 1.07	(Δ/σ)_max_ < 0.001
3216 reflections	Δρ_max_ = 0.23 e Å^−^^3^
177 parameters	Δρ_min_ = −0.30 e Å^−^^3^
1 restraint	Absolute structure: Flack (1983), 1368 Friedel pairs
Primary atom site location: structure-invariant direct methods	Flack parameter: −0.1 (7)

### Special details

**Table:**

Experimental. The crystal was placed in the cold stream of an Oxford Cryosystems Cobra open-flow nitrogen cryostat (Cosier & Glazer, 1986) operating at 100.0 (1) K.
Geometry. All e.s.d.'s (except the e.s.d. in the dihedral angle between two l.s. planes) are estimated using the full covariance matrix. The cell e.s.d.'s are taken into account individually in the estimation of e.s.d.'s in distances, angles and torsion angles; correlations between e.s.d.'s in cell parameters are only used when they are defined by crystal symmetry. An approximate (isotropic) treatment of cell e.s.d.'s is used for estimating e.s.d.'s involving l.s. planes.
Refinement. Refinement of *F*^2^ against ALL reflections. The weighted *R*-factor *wR* and goodness of fit *S* are based on *F*^2^, conventional *R*-factors *R* are based on *F*, with *F* set to zero for negative *F*^2^. The threshold expression of *F*^2^ > σ(*F*^2^) is used only for calculating *R*-factors(gt) *etc*. and is not relevant to the choice of reflections for refinement. *R*-factors based on *F*^2^ are statistically about twice as large as those based on *F*, and *R*- factors based on ALL data will be even larger.

### Fractional atomic coordinates and isotropic or equivalent isotropic displacement parameters (Å^2^)

**Table:**

	*x*	*y*	*z*	*U*_iso_*/*U*_eq_	Occ. (<1)
F1	0.5951 (6)	−0.360 (3)	0.6163 (9)	0.0636 (17)	0.505 (17)
F2	0.5409 (3)	0.0250 (13)	0.6382 (9)	0.075 (2)	0.505 (17)
F3	0.5859 (5)	−0.076 (4)	0.4723 (7)	0.104 (4)	0.505 (17)
F1X	0.6094 (7)	−0.352 (2)	0.5755 (15)	0.097 (4)	0.495 (17)
F2X	0.5478 (4)	−0.032 (2)	0.6626 (5)	0.080 (2)	0.495 (17)
F3X	0.5752 (3)	0.0168 (15)	0.4869 (6)	0.0479 (14)	0.495 (17)
O1	0.67666 (7)	0.0887 (3)	0.73348 (10)	0.0384 (3)	
O2	0.70767 (6)	0.1522 (3)	0.54434 (9)	0.0312 (3)	
N1	0.80834 (7)	0.5530 (3)	0.60374 (10)	0.0248 (3)	
N2	0.78211 (9)	0.5050 (4)	0.80182 (12)	0.0320 (3)	
C1	0.81952 (8)	0.6336 (4)	0.71687 (12)	0.0257 (3)	
C2	0.87136 (9)	0.8495 (4)	0.73742 (14)	0.0307 (3)	
H2A	0.8806	0.9141	0.8156	0.037*	
C3	0.90806 (9)	0.9649 (4)	0.64496 (15)	0.0316 (3)	
H3A	0.9432	1.1084	0.6599	0.038*	
C4	0.89527 (8)	0.8768 (4)	0.52703 (13)	0.0279 (3)	
C5	0.84486 (8)	0.6703 (4)	0.51114 (12)	0.0257 (3)	
H5A	0.8347	0.6054	0.4333	0.031*	
C6	0.93586 (10)	1.0012 (4)	0.42471 (17)	0.0364 (4)	
H6A	0.9186	0.9147	0.3510	0.055*	
H6B	0.9868	0.9594	0.4342	0.055*	
H6C	0.9286	1.2110	0.4221	0.055*	
C7	0.59832 (9)	−0.0794 (4)	0.58757 (15)	0.0312 (3)	
C8	0.66781 (9)	0.0707 (4)	0.62616 (13)	0.0268 (3)	
H2N2	0.7456 (13)	0.369 (6)	0.785 (2)	0.040 (6)*	
H1N2	0.7919 (14)	0.558 (6)	0.873 (3)	0.050 (7)*	
H1N1	0.7723 (13)	0.405 (6)	0.585 (2)	0.043 (6)*	

### Atomic displacement parameters (Å^2^)

**Table:**

	*U*^11^	*U*^22^	*U*^33^	*U*^12^	*U*^13^	*U*^23^
F1	0.075 (3)	0.027 (2)	0.089 (4)	−0.0046 (19)	−0.017 (2)	0.007 (2)
F2	0.0328 (17)	0.044 (2)	0.148 (6)	0.0071 (15)	0.004 (3)	−0.043 (3)
F3	0.093 (4)	0.194 (10)	0.0252 (15)	−0.097 (5)	−0.010 (2)	0.014 (4)
F1X	0.092 (6)	0.021 (2)	0.177 (11)	0.005 (3)	−0.069 (7)	−0.017 (5)
F2X	0.041 (3)	0.154 (6)	0.044 (2)	−0.038 (3)	0.0180 (16)	−0.011 (3)
F3X	0.0410 (16)	0.060 (3)	0.042 (3)	−0.0108 (16)	−0.0251 (16)	0.0201 (19)
O1	0.0462 (7)	0.0534 (9)	0.0156 (5)	−0.0029 (6)	0.0001 (4)	0.0020 (5)
O2	0.0337 (5)	0.0450 (7)	0.0149 (4)	−0.0038 (5)	0.0021 (4)	−0.0058 (5)
N1	0.0316 (6)	0.0292 (7)	0.0136 (5)	0.0038 (5)	−0.0025 (4)	−0.0004 (5)
N2	0.0454 (8)	0.0368 (9)	0.0138 (5)	0.0023 (7)	0.0001 (5)	−0.0032 (5)
C1	0.0341 (7)	0.0277 (8)	0.0154 (6)	0.0089 (6)	−0.0027 (5)	−0.0032 (6)
C2	0.0412 (8)	0.0290 (9)	0.0220 (6)	0.0061 (7)	−0.0067 (6)	−0.0064 (6)
C3	0.0351 (8)	0.0299 (9)	0.0298 (7)	0.0036 (6)	−0.0051 (6)	−0.0059 (7)
C4	0.0305 (7)	0.0298 (9)	0.0235 (7)	0.0075 (6)	−0.0013 (5)	0.0003 (6)
C5	0.0321 (6)	0.0302 (8)	0.0147 (5)	0.0069 (6)	−0.0025 (5)	−0.0014 (5)
C6	0.0394 (8)	0.0377 (10)	0.0320 (7)	0.0008 (7)	0.0038 (7)	0.0033 (8)
C7	0.0380 (7)	0.0299 (9)	0.0255 (6)	−0.0003 (6)	−0.0007 (6)	0.0037 (6)
C8	0.0318 (7)	0.0305 (8)	0.0181 (6)	0.0052 (6)	−0.0001 (5)	−0.0008 (6)

### Geometric parameters (Å, º)

**Table:**

F1—C7	1.341 (11)	N2—H1N2	0.86 (3)
F2—C7	1.311 (6)	C1—C2	1.412 (2)
F3—C7	1.325 (8)	C2—C3	1.361 (3)
F1X—C7	1.287 (11)	C2—H2A	0.9500
F2X—C7	1.290 (5)	C3—C4	1.416 (2)
F3X—C7	1.297 (6)	C3—H3A	0.9500
O1—C8	1.2289 (18)	C4—C5	1.355 (2)
O2—C8	1.2478 (19)	C4—C6	1.500 (2)
N1—C1	1.3500 (18)	C5—H5A	0.9500
N1—C5	1.3640 (19)	C6—H6A	0.9800
N1—H1N1	0.98 (3)	C6—H6B	0.9800
N2—C1	1.330 (2)	C6—H6C	0.9800
N2—H2N2	0.95 (3)	C7—C8	1.538 (2)
			
C1—N1—C5	122.78 (14)	C4—C6—H6A	109.5
C1—N1—H1N1	119.9 (15)	C4—C6—H6B	109.5
C5—N1—H1N1	117.3 (15)	H6A—C6—H6B	109.5
C1—N2—H2N2	121.8 (14)	C4—C6—H6C	109.5
C1—N2—H1N2	116.0 (19)	H6A—C6—H6C	109.5
H2N2—N2—H1N2	122 (2)	H6B—C6—H6C	109.5
N2—C1—N1	118.73 (15)	F1X—C7—F2X	110.9 (7)
N2—C1—C2	124.01 (14)	F1X—C7—F3X	107.3 (7)
N1—C1—C2	117.26 (14)	F2X—C7—F3X	106.0 (5)
C3—C2—C1	119.86 (14)	F2—C7—F1	102.4 (6)
C3—C2—H2A	120.1	F3—C7—F1	104.0 (8)
C1—C2—H2A	120.1	F1X—C7—C8	109.7 (5)
C2—C3—C4	121.77 (16)	F2X—C7—C8	110.8 (3)
C2—C3—H3A	119.1	F3X—C7—C8	112.1 (3)
C4—C3—H3A	119.1	F2—C7—C8	113.8 (3)
C5—C4—C3	116.49 (14)	F3—C7—C8	115.0 (4)
C5—C4—C6	121.40 (14)	F1—C7—C8	114.0 (5)
C3—C4—C6	122.10 (16)	O1—C8—O2	129.24 (16)
C4—C5—N1	121.84 (13)	O1—C8—C7	115.18 (15)
C4—C5—H5A	119.1	O2—C8—C7	115.57 (13)
N1—C5—H5A	119.1		
			
C5—N1—C1—N2	−179.15 (15)	F2X—C7—C8—O1	32.3 (6)
C5—N1—C1—C2	0.2 (2)	F3X—C7—C8—O1	150.5 (4)
N2—C1—C2—C3	178.70 (16)	F2—C7—C8—O1	51.3 (5)
N1—C1—C2—C3	−0.6 (2)	F3—C7—C8—O1	174.4 (9)
C1—C2—C3—C4	0.7 (2)	F1—C7—C8—O1	−65.7 (5)
C2—C3—C4—C5	−0.4 (2)	F1X—C7—C8—O2	88.4 (9)
C2—C3—C4—C6	−179.53 (17)	F2X—C7—C8—O2	−148.9 (5)
C3—C4—C5—N1	−0.1 (2)	F3X—C7—C8—O2	−30.7 (4)
C6—C4—C5—N1	179.11 (15)	F2—C7—C8—O2	−129.9 (5)
C1—N1—C5—C4	0.1 (2)	F3—C7—C8—O2	−6.8 (9)
F1X—C7—C8—O1	−90.5 (9)	F1—C7—C8—O2	113.2 (5)

### Hydrogen-bond geometry (Å, º)

**Table:**

*D*—H···*A*	*D*—H	H···*A*	*D*···*A*	*D*—H···*A*
N1—H1*N*1···O2	0.98 (3)	1.75 (3)	2.7281 (19)	177 (2)
N2—H2*N*2···O1	0.95 (3)	1.92 (3)	2.865 (2)	173 (2)
N2—H1*N*2···O2^i^	0.86 (3)	1.99 (3)	2.8347 (18)	167 (3)
C3—H3*A*···F2^ii^	0.95	2.51	3.429 (6)	164
C5—H5*A*···O1^iii^	0.95	2.27	3.1910 (19)	162

Symmetry codes: (i) −*x*+3/2, *y*+1/2, *z*+1/2; (ii) *x*+1/2, −*y*+3/2, *z*; (iii) −*x*+3/2, *y*+1/2, *z*−1/2.
